# Bacterial vaginosis, human papilloma virus and herpes viridae do not predict vaginal HIV RNA shedding in women living with HIV in Denmark

**DOI:** 10.1186/s12879-017-2477-7

**Published:** 2017-05-31

**Authors:** Maria Wessman, Kristina Thorsteinsson, Jørgen S. Jensen, Merete Storgaard, Frederikke F. Rönsholt, Isik S. Johansen, Gitte Pedersen, Lars Nørregård Nielsen, Jesper Bonde, Terese L. Katzenstein, Nina Weis, Anne-Mette Lebech

**Affiliations:** 1 Department of Infectious Diseases, Copenhagen University Hospital, Kettegaard Allé 30, Hvidovre, DK-2650 Copenhagen, Denmark; 2 Department of Microbiology and Infection Control, Statens Serum Institut, 2300 Copenhagen, Artillerivej 5 Denmark; 3 Department of Infectious Diseases, Aarhus University Hospital, Palle Juul-Jensens Boulevard 99, Skejby, 8200 Aarhus, Denmark; 4 Department of Infectious Diseases, Copenhagen University Hospital, Blegdamsvej 9, Rigshospitalet, 2100 Copenhagen, Denmark; 5 Department of Infectious Diseases, Odense University Hospital, Søndre Boulevard 29, 5000 Odense, Denmark; 6 Department of Infectious Diseases, Aalborg University Hospital, Hobrovej 18-22, 9100 Aalborg, Denmark; 7 Department of Lung- and Infectious Diseases, Nordsjællands Hospital, Dyrehavevej 29, 3400 Hillerød, Denmark; 8 Department of Pathology, Copenhagen University Hospital, Kettegaard Allé 30, Hvidovre, -2650 Copenhagen, DK Denmark; 9 Clinical Research Centre, Copenhagen University Hospital, Hvidovre, Copenhagen, Denmark; 10 Department of Clinical Medicine, Faculty of Health and Medical Sciences, University of Copenhagen, Copenhagen, Denmark

**Keywords:** Women living with HIV, Bacterial vaginosis, HIV RNA vaginal shedding, Herpes viridae, Human papilloma virus

## Abstract

**Background:**

Bacterial vaginosis (BV) has been found to be associated with HIV acquisition and transmission.

This is suggested to be due to higher HIV RNA levels in cervicovaginal fluids in women living with HIV (WLWH) with BV, as bacteria associated with BV may induce viral replication and shedding in the genital tract despite undetectable HIV RNA plasma viral load.

We examined the prevalence and diagnostic predictors of BV and HIV-1 RNA vaginal shedding in women living with HIV (WLWH) in Denmark, taking into account the presence of human papillomavirus (HPV) and herpes viridae.

**Methods:**

WLWH between 18–51 years were recruited from six Departments of Infectious Diseases in Denmark during enrolment in the SHADE cohort; a prospective cohort study of WLWH attending regular outpatient care. BV was diagnosed by microscopy of vaginal swabs and PCR was used for detection of BV-associated bacteria, HPV, herpes viridae, and vaginal HIV viral load.

**Results:**

Median age of the 150 included women was 41 years; ethnicity was predominantly White (35%) or Black (47%). The majority (96%) was on ART and had undetectable (85%) plasma HIV RNA (<40 copies/mL).

BV was diagnosed in 32%. Overall, 11% had detectable vaginal HIV RNA. Both before and after adjustment for BV, age, ethnicity, plasma HIV RNA, CD4 cell count, herpes viridae and HPV, we found no significant predictors of HIV RNA vaginal shedding.

**Conclusion:**

In well-treated WLWH, BV, herpes viridae or HPV do not predict vaginal HIV RNA shedding. This implies that HIV shedding does not seem to be increased by BV.

## Background

Bacterial vaginosis (BV) is characterized by an overgrowth of anaerobic bacteria and a relative loss of lactobacillus species. This leads to an increase in vaginal pH and a malodorous vaginal discharge [1]. Bacterial vaginosis is associated with complications such as miscarriage, premature birth, pelvic inflammatory disease and tubal factor infertility [2].

Studies have shown a high incidence of BV among women living with Human Immunodeficiency Virus-1 (WLWH) (HIV) and an increased risk of both HIV acquisition and HIV transmission in women with BV [3, 4].

The association between BV and an increased rate of HIV transmission has been suggested to include several mechanisms; a higher HIV RNA concentration has been found in cervicovaginal fluids in WLWH with BV, [5] and bacteria associated with BV may induce viral replication and shedding in the genital tract [6]. Both components may lead to increased HIV transmission [4]. A study from sub-Saharan Africa showed that BV was associated with a three-fold higher risk of HIV female-to-male transmission among sero-discordant couples [4]. However, in most conducted studies, WLWH did not receive antiretroviral treatment (ART) which reduces the HIV transmission significantly [7].

On the other hand, Alcaide et al. [8] showed that in 128 Zambian WLWH on ART, plasma HIV RNA levels were significantly associated with vaginal HIV RNA shedding, while BV was not.

The aim of the study was to investigate the prevalence of BV and vaginal HIV RNA shedding and to identify diagnostic predictors, including herpes viridae and human papilloma virus (HPV), of BV and HIV RNA vaginal shedding in predominantly well-treated WLWH.

## Methods

### Study setting

Denmark has a population of 5.6 million inhabitants, with an estimated 5000 HIV-infected individuals of which 1400 are women [9]. Annually, 250–300 individuals are diagnosed with HIV [10]. Medical care, including ART, is provided free of charge [11]. WLWH >18 years were recruited from six Departments of Infectious Diseases in Denmark (Copenhagen University Hospitals, Hvidovre and Rigshospitalet, Aarhus University Hospital, Skejby, Odense University Hospital, Nordsjællands Hospital and Aalborg University Hospital) during enrolment in the SHADE (Study on HIV, cervical Abnormalities and infections in women in Denmark) [12]. The SHADE cohort is a prospective cohort study of WLWH in Denmark [12]. A cut-off age of 51 years was chosen as the median age for menopause is 51 years and a microscopic BV diagnosis is questionable in post-menopausal women [13]. Exclusion criterion was pregnancy. Collection of samples took place between February 2013 and March 2014.

### Danish HIV cohort study

The Danish HIV Cohort Study is a nationwide, prospective, observational, multicentre, population-based cohort study of all People Living with HIV (PLWH) seen at Danish Departments of Infectious Diseases since 1 January 1995 [11]. The database is updated annually and contains extensive data on PLWH, e.g. demographic data, date of HIV diagnosis, ART regimen, CD4 counts and HIV RNA levels [11].

### The civil registration system

The Civil Registrations System is a national registry of all Danish residents [14]. At birth or immigration a unique, 10-digit personal identification number (PIN) is assigned to each individual. The PIN was used to link the Danish HIV Cohort Study to the SHADE cohort.

### Ethical statement

The study was approved by the Danish Data Protection Agency (2015–231-0126, 2012–58-0004 and 2012–41-0005) and the Danish Regional Committee on Health Research Ethics (H-3-2010-119 and H-2-2014-102). All patients signed informed consent.

### Microscopy - detection of bacterial vaginosis

Vaginal swabs were taken and a flocked swab was collected in Universal Transport Medium (Copan, Brescia, Italy) and sent to Statens Serum Institut, Copenhagen. The smears were Gram stained and slides were evaluated by the same observer. Each sample was scored on an average of at least five field views according to the Nugent score: Grade I (score 0–3): normal flora; grade II (score 4–6): intermediary stage and grade III (score 7–10): BV. [1] The microscopy diagnosis is used in the statistical analyses, as Nugent’s scoring system is considered gold standard.

### PCR – Detection of bacterial vaginosis

Quantitative real-time polymerase chain reaction (qPCR) of vaginal swabs for detection of: *Atopobium vaginae*, *Megasphaera type 1*, *Bacterial Vaginosis Associated Bacterium type 1* (BVAB1) and *Prevotella spp.* was carried out as previously described [15]. The bacterium BVAB1 was used as it has been shown to be significantly associated with high Nugent scores [16]. The remaining three bacteria were used as they previously have been shown to have good sensitivity and specificity in PCR analysis [15, 17, 18].

When at least one of the abovementioned four bacteria was above the cut-off value, the test was considered positive for BV. Cut-off values for the four bacteria were used as described by Datcu et al. [15].

### PCR - detection of vaginal HIV RNA

Vaginal swabs were collected and placed in 3 mL Copan Universal Transport medium (Copan, Brescia, Italy) and frozen at −80 °C. Between 0.3 mL - 1.0 mL was available from each swab. Samples were thawed at room temperature, thoroughly vortexed and centrifuged for one hour at 17,000 G. The pellet was dissolved in 1.0 mL phosphate-buffered saline. HIV RNA was measured using AmpliPrep (Roche Molecular Systems, Inc.) and COBAS TaqMan 48 (COBAS TaqMan HIV test, v2.0, Roche Molecular Systems, Inc.). Results are presented as copies HIV RNA/swab. Detection limit for the COBAS TaqMan is 20 copies/mL and lowest detected level in our samples was ≥66 copies/swab.

The test was validated prior to study analysis; 10 samples of Copan Universal Transport medium with a known concentration of HIV RNA were analysed using the same procedure as recommended by the manufacturer – validating the test for use in this medium.

### PCR - human papillomavirus

Cervical samples were examined for 35 defined high and low risk HPV by the CLART HPV2 assay (Genomica, Madrid, Spain). The assay detects genotype specific HPV L1 fragments from HPV6, 11, 16, 18, 26, 31, 33, 35, 39, 40, 42, 43, 44, 45, 51, 52, 53, 54, 56, 58, 59, 61, 62, 66, 68, 70, 71, 72, 73, 81, 82, 83, 84, 85 and 89 [19]. Analytical DNA extractions were conducted using the MagNAPure LC96 (LC Total Nuclei Acid kit, Roche Molecular Systems, Rotkreuz, Switzerland).

### PCR – herpes viridae

Diagnostics on vaginal swabs was conducted using the Entherpex PCR microarray system in concordance with manufacturer’s specifications (Genomica, Madrid, Spain). The array simultaneously detects: Herpes simplex virus 1 and 2, Cytomegalovirus, Epstein-Barr virus, Human herpes virus 6 and 7, and Varicella zoster virus.

Both HPV and herpes virus analyses were done at the Department of Pathology, Copenhagen University Hospital, Hvidovre, Denmark.

### Statistical analyses

Categorical variables were reported as counts and percentages and compared by chi-square test or Fisher’s exact test, as appropriate. Continuous variables were summarized as median and interquartile ranges (IQR) and compared using the Wilcoxon rank sum test. Uni- and multiple logistic regression analyses were performed to identify predictors of HIV RNA vaginal shedding and BV. Odds ratios (ORs) and 95% confidence intervals were estimated and adjusted for candidate predictor variables chosen a priori; age at inclusion (<40 versus ≥40 years), ethnicity, plasma HIV RNA (<40 versus ≥40 copies/mL), CD4 count (<200, ≥200–349 and ≥350 cells/μL), herpes PCR positive, HPV PCR positive, and BV (positive by microscopy). The validity of the model was tested using the Hosmer and Lemeshow Goodness-of-Fit Test. Individuals with missing explanatory values were excluded from the multiple regression analyses, i.e. considered missing at random. SAS statistical software version 9.3 (SAS Institute Inc., Cary, NC, USA) was used for data analysis and *p*-values below 0.05 (two-sided) were considered statistically significant.

Results of BV were statistically analysed using Nugent score, as this is the gold standard of BV diagnosis [1].

## Results

### Demographics (Table 1)

Two hundred thirty-four WLWH were eligible for inclusion [12]. Of these, 34 were excluded as samples for microscopy (*n* = 3) or vaginal HIV RNA (*n* = 31) analyses were missing and 50 women were excluded as they were 51 years or older, leaving 150 women included in the study. Table 1 shows the baseline demographic characteristics. The median age was 41 years and ethnicity was predominantly Black (47%) or White (35%). The vast majority of women were on ART and 85% had undetectable plasma HIV RNA (<40 copies/mL).

**Table 1:** Baseline characteristics of 150 women living with HIV, included in the study

Demographic data	Study Participants
Duration of HIV infection (years), median (IQR)	11.3 (6.6–16.1)
Age at inclusion (years), median (IQR)	41.1 (36.5–45.5)
Ethnicity, *n* (%)	
Black	71 (47.3)
White	53 (35.3)
Asian	24 (16.0)
Other	2 (1.3)
Place of HIV transmission, *n* (%)	
Denmark	45 (32.4)
Europe + US	13 (9.4)
Africa	61 (43.9)
Asia	20 (14.4)
(missing)	(11)
Mode of transmission, *n* (%)	
Heterosexual	137 (93.8)
IDU^a^	4 (2.7)
Other	5 (3.4)
(missing)	(4)
CD4 count (cells/μl), *n* (%)	
< 200	4 (2.9)
200–350	18 (13.0)
> 350	118 (84.1)
(missing)	(11)
ART, *n* (%)	
Yes	144 (96.0)
No	6 (4.0)
ART groups, *n* (%)	
2 NRTI’s + 1 NNRTI	59 (50.0)
2 NRTI’s + PI’s	65 (45.1)
Other regimen	20 (13.9)
AIDS prior to inclusion *n* (%)	
Yes	20 (13.3)
No	130 (86.7)
Number of lifetime sexual partners	
< 5	38 (25.3)
5–14	62 (41.3)
15–25	24 (16.0)
> 25	25 (16.7)
Does not want to respond	1 (0.7)
Current use of contraception *n* (%)	
Condom Yes	67 (44.7)
Condom No	83 (55.3)
Symptoms^b^ from the lower abdomen	
Yes	35 (23.3)
No	115 (76.7)

ART groups: *NRTI* Nucleoside Reverse Transcriptase Inhibitors, *NNRTI* Non-Nucleoside Reverse Transcriptase Inhibitors, *PI* Protease Inhibitors

^a^ *IDU* Intravenous Drug Use

^b^Symptoms included: increased vaginal discharge, disturbing vaginal smell, burning sensation upon urination, bleeding disturbances, bleeding during intercourse, pain during intercourse, general pain and “other”

Ninety-four percent were heterosexually infected, most had <15 lifetime sexual partners. Symptoms from the lower abdomen were reported by 23% of the women. Most commonly reported symptoms included increased vaginal discharge, bleeding disturbances and pain during intercourse.

### Bacterial vaginosis and vaginal HIV RNA shedding (Table 2)

Table 2 shows that Nugent grade III was found in 32% of the women, grade II in 37% and grade I in 31%. When microscopy and PCR were combined by including all women with grade III together with any BV associated bacterium present above threshold, 40% of the women were positive for BV. Also shown in Table 2 are the results of the four bacteria associated with BV, analysed with PCR. There was no statistically significant association between HIV RNA vaginal shedding and BV diagnosed with microscopy or PCR. The only statistically significant finding in the analysis was being PCR negative for *Prevotella spp*. (*p* = 0.038).

**Table 2:** Prevalence of Bacterial Vaginosis, vaginal HIV RNA shedding and plasma HIV RNA viral load in 150 women living with HIV included in the study

Diagnosis	Study Participants *n* (% of all participants)	Positive for vaginal HIV RNA shedding *n* (% of total)	Combined *p*-value
Bacterial vaginosis (BV), Nugent score^a^			
Grade I	47 (31.3)	5 (10.6)	-
Grade II	55 (36.7)	7 (12.7)	-
Grade III	48 (32.0)	5 (10.4)	0.92
Bacterial vaginosis (PCR)			
Atopobium Vaginae positive	44 (30.1)	5 (11.4)	-
Atopobium Vaginae negative	102 (69.9)	12 (11.8)	0.94
(missing)	(4)		
Bacterial vaginosis (PCR)			
Megasphaera positive	34 (22.8)	3 (8.8)	-
Megasphaera negative	115 (77.2)	14 (12.2)	0.59
(missing)	(1)		
Bacterial vaginosis (PCR)			
BVAB1 positive	9 (6.1)	1 (11.1)	-
BVAB1 negative	139 (93.9)	15 (10.8)	0.98
(missing)	(2)		
Bacterial vaginosis (PCR)			
Prevotella positive	40 (26.9)	1 (2.5)	-
Prevotella negative	109 (73.1)	16 (14.7)	0.038
(missing)	(1)		
Bacterial vaginosis (PCR)			
At least one bacteria positive with PCR	54 (36.0)	5 (9.3)	-
All bacteria negative with PCR	96 (64.0)	12 (12.5)	0.55
Combined PCR and microscopy			
Positive	60 (40.0)	6 (10.0)	-
Negative	90 (60.0)	11 (12.2)	0.67
BV positive, by ethnicity			
White	22 (45.8)	6 (11.3)	-
Black	18 (37.5)	1 (4.2)	-
Asian	7 (14.6)	10 (14.1)	-
Other	1 (2.1)	0 (0)	0.59
Vaginal HIV RNA			
Detectable ≥66 copies/swab)	17 (11.3)	-	-
Not detectable (<66 copies/swab)	133 (88.7)	-	-
Plasma HIV RNA < 40 copies/mL			
Detectable	21 (15.2)	2 (1.5)	-
Not detectable	117 (84.8)	11 (9.4)	1.0
(missing)	(12)		

^a^ Nugent score Grade I = normal flora, Grade II = intermediate flora, Grade III = Bacterial vaginosis

There was no significant difference in BV diagnosis between women of White and Black ethnicity (*p* = 0.21). There was no difference in reported symptoms from the lower abdomen or number of lifetime sexual partners according to BV status. Further, we found no correlation between BV status and detectable plasma HIV RNA. Treatment with protease inhibitors (PIs) was not associated with BV (*p* = 0.28) but non-nucleoside reverse transcriptase inhibitor (NNRTI) treatment was positively associated with BV (*p* = 0.047). Seventy-five WLWH received PIs and of those, 20 had BV while 62 WLWH received NNRTI’s and of those 25 had BV.

### Predictors of vaginal HIV RNA shedding (Table 3)

The range for detectable HIV RNA in vaginal swabs was 66–31,650 copies/swab with a median value of 191 copies/swab. Overall, 11% of the women had detectable vaginal HIV RNA (≥66 copies/swab). Of these, 94% were on ART. Two women that were positive for vaginal HIV RNA shedding, had detectable plasma HIV RNA levels (> 40 copies/mL), for three women the plasma levels were missing. Of the 11% of women positive for vaginal HIV RNA shedding, 29% had BV. There was no significant association between ART regimen and vaginal HIV RNA shedding.

**Table 3:** Predictors for vaginal HIV RNA shedding in 150 women living with HIV, included in the study. Unadjusted and adjusted for: age at inclusion, ethnicity, plasma HIV RNA, CD4 cell count, herpes viridae DNA, HPV and bacterial vaginosis (Nugent score)

Predictors of vaginal HIV RNA^a^	Vaginal HIV RNA shedding positive (*n* = 17)	Vaginal HIV RNA shedding negative (*n* = 133)	Unadjusted odds ratios	*p*-value	Adjusted odds ratios	*p*-value
Age at inclusion (years), *n* (%)						
< 40	6 (9.8)	55 (90.2)	1.00	-	1.00	-
≥ 40	11 (12.4)	78 (87.6)	1.29 (0.45–3.71)	0.63	1.43 (0.34–6.00)	0.63
Ethnicity, *n* (%)						
White	6 (11.3)	47 (88.7)	1.00	-	1.00	-
Asian	1 (4.2)	23 (95.8)	0.34 (0.04–3.0)	0.33	0.67 (0.06–7.27)	0.74
Black	10 (14.1)	61 (85.9)	1.28 (0.44–3.79)	0.65	1.70 (0.41–7.16)	0.47
(missing)	(0)	(2)				
Combined *p*-value				0.73		0.60
HIV RNA (copies/ml), *n* (%)						
≥ 40	2 (9.5)	19 (90.5)	1.00	-	1.00	-
< 40	11 (9.4)	106 (90.6)	0.99 (0.20–4.80)	0.99	1.19 (0.21–6.63)	0.85
(missing)	(4)	(8)				
CD4 count (cells/μl), *n* (%)						
< 350	2 (9.1)	20 (90.9)	1.00	-	1.00	-
≥ 350	11 (9.4)	106 (90.6)	1.04 (0.21–5.04)	0.96	1.15 (0.22–6.09)	0.87
(missing)	(4)	(7)				
Herpes viridae PCR positive, *n* (%)						
Yes	7 (13.5)	45 (86.5)	1.00	-	1.00	-
No	9 (9.6)	85 (90.4)	0.68 (0.24–1.95)	0.47	0.83 (0.21–3.26)	0.79
(missing)	(1)	(3)				
HPV^b^ PCR positive, *n* (%)						
Yes	10 (14.9)	57 (85.1)	1.00	-	1.00	-
No	6 (8.8)	62 (91.2)	0.55 (0.19–1.62)	0.23	0.73 (0.21–2.60)	0.63
(missing)	(1)	(14)				
BV^c^ by Nugent score, *n* (%)						
Yes	5 (10.4)	43 (89.4)	1.00	-	1.00	-
No	12 (11.8)	90 (88.2)	1.15 (0.38–3.46)	0.81	1.80 (0.43–7.62)	0.42

^a^ The validity of the model was tested using the Hosmer and Lemeshow Goodness-of-Fit Test

^b^ *HPV* human papilloma virus

^c^ Bacterial vaginosis

There was no difference in vaginal HIV RNA shedding according to BV status (*p* = 0.21). None of the chosen variables predicted vaginal HIV RNA shedding in the adjusted analyses. The Hosmer and Lemeshow Goodness-of-Fit tests demonstrated sufficient fit (*p =* 0.097, *p =* 0.29).

### Predictors of bacterial vaginosis (Table 4)

Table 4 shows predictors of bacterial vaginosis. When analyzing age at inclusion, ethnicity, plasma viral load, CD4 cell counts, herpes viridae, HPV or vaginal HIV RNA shedding, no significant predictors of BV were found, apart from suppressed viral load (*p* = 0.05).

**Table 4:** Table over predictors of bacterial vaginosis in 150 women living with HIV, included in the study. Unadjusted and adjusted for: age at inclusion, ethnicity, plasma HIV RNA, CD4 cell count, herpes viridae, human papilloma virus and vaginal HIV RNA shedding

Predictors of Bacterial Vaginosis^a^	BV^b^ positive (*n* = 48)	BV negative (*n* = 102)	Unadjusted odds ratios	*p*-value	Adjusted odds ratios	*p*-value
Age at inclusion (years), *n* (%)						
< 40	23 (37.7)	38 (62.3)	1.00	-	1.00	-
≥ 40	25 (28.1)	64 (71.9)	0.65 (0.32–1.29)	0.22	0.63 (0.26–1.49)	0.29
Ethnicity, *n* (%)						
White	22 (41.5)	31 (58.5)	1.00	-	1.00	-
Asian	7 (29.2)	17 (70.8)	0.58 (0.21–1.64)	0.30	0.36 (0.10–1.29)	0.12
Black	18 (25.4)	53 (74.6)		0.06		0.06
(missing)	(1)	(1)	0.48 (0.22–1.03)		0.42 (0.17–1.03)	
Combined *p*-value				0.16		0.11
Plasma HIV RNA (copies/ml), *n* (%)						
≥ 40	3 (14.3)	18 (85.7)	1.00	-	1.00	-
< 40	40 (34.2)	77 (65.8)	3.12 (0.87–11.22)	0.082	4.0 (1.02–15.65)	0.05
(missing)	(5)	(7)				
CD4 count (cells/μl), *n* (%)						
< 350	4 (18.2)	18 (81.8)	1.00	-	1.00	-
≥ 350	39 (33.3)	78 (66.7)	2.25 (0.71–7.10)	0.17	2.04 (0.61–6.83)	0.25
(missing)	(5)	(6)				
Herpes PCR positive, *n* (%)						
Yes	18 (34.6)	34 (65.4)	1.00	-	1.00	-
No	28 (29.8)	66 (70.2)	0.80 (0.39–1.65)	0.55	1.08 (0.43–2.70)	0.87
(missing)	(2)	(2)				
HPV^c^ PCR positive, *n* (%)						
Yes	23 (34.3)	44 (65.7)	1.00	-	1.00	-
No	19 (27.9)	49 (72.1)	0.74 (0.36–1.54)	0.42	0.88 (0.39–2.00)	0.76
(missing)	(6)	(9)				
Vaginal HIV RNA shedding positive, *n* (%)						
Yes	5 (29.4)	12 (70.6)	1.00	-	1.00	-
No	43 (32.3)	90 (67.7)	1.15 (0.38–3.46)	0.81	1.11 (0.26–4.84)	0.89

^a^ The validity of the model was tested using the Hosmer and Lemeshow Goodness-of-Fit Test

^b^ *BV* Bacterial Vaginosis

^c^ *HPV* Human Papilloma Virus

## Discussion

This study examined the prevalence and diagnostic predictors of BV and HIV RNA vaginal shedding in Danish WLWH, while also taking into account HPV and herpes viridae.

In this nationwide study of 150 predominantly well-treated WLWH the overall BV prevalence by Nugent score was 32% and HIV RNA was detected in 11% of vaginal swabs. However, no predictors of HIV RNA vaginal shedding were found, including being BV positive. Furthermore, the presence of vaginal herpes viridae and/or HPV or vaginal HIV RNA shedding did not predict BV.

### Bacterial vaginosis and vaginal HIV RNA shedding

Similar to our results, a study among 311 American WLWH aged 18–45 years, found a BV prevalence (Nugent’s criteria used for diagnosis) of 36% [20]. Notably, almost half of the study participants were not receiving ART, more than two-thirds had CD4 cell counts below 500 cells/mm^3^ and the majority were of Black ethnicity [20]. Despite the higher rate of well- treated women in the present study, we found a comparable BV prevalence, confirming that BV is highly prevalent, irrespective of ART. In comparison, a Danish study of the general population found that 16% of 880 women aged 15–45 years had BV according to Nugent’s criteria [21]. Women were included from one general practice, where they attended the clinic due to abnormal vaginal discharge, other genito-urinary symptoms or for a routine check-up [21]. On the other hand, a study from a Sexually Transmitted Infections (STI’s) clinic in Copenhagen where 76 women with symptoms from the lower abdomen were examined for STI’s, found BV to be the leading cause of vaginal discharge with a prevalence of 62% (method of diagnosis not specified) [22]. Women in our study, however, were examined regardless of symptoms, which might explain the lower occurrence of BV compared to women attending an STI clinic with complaints of symptoms from the lower abdomen. However, our study population had double the amount of positive BV smears compared to a sample from the general population, which may be explained by the presence of HIV infection.

It has previously been shown that women of Black ethnicity have a higher prevalence of BV (diagnosed with Amsel’s criteria), compared to women of White ethnicity [23]. However, in the present study, no difference in BV between women of Black and White ethnicity was found. The lack of difference between the two populations may be due to the small sample size or different cultural practices in a Danish setting, compared to American or sub-Saharan settings [24, 25].

Molecular methods for detection of BV-associated bacteria were used in order to be able to also classify women with intermediate (Nugent II) flora and with an aim to sub-classify BV according to the dominating bacterial composition. However, neither the classification according to presence of bacterial loads above threshold, nor the stratification according to the individual BV-defining species showed any correlation with HIV shedding.

We found a vaginal HIV RNA prevalence of 11%. Similarly, Neely et al. [26] detected HIV RNA in 15% of cervical swabs obtained from 290 well treated WLWH. The authors found that shedding was associated with NNRTI use versus PI use and illicit drug use, while no correlation was found between HIV RNA shedding and BV, as more non-shedders than shedders were diagnosed with BV (21% versus 16%) [26]. We found no association between PIs, NNRTIs and vaginal HIV RNA shedding. However, BV was associated with NNRTI use. The reason for this association may be spurious, although NNRTIs seem to have lower concentration in genital fluids compared to PIs [27]. Though vaginal HIV RNA shedding has been shown to correlate with plasma HIV RNA, a separate reservoir of vaginal HIV RNA has also been described [20].

Due to a lack of statistical power (12 women on hormonal contraceptives and one with vaginal HIV RNA shedding) hormonal contraception was not included in the analyses as a possible predictor of vaginal HIV RNA shedding in our study.

### Predictors of bacterial vaginosis and vaginal HIV RNA shedding

We found no statistically significant association between HIV RNA vaginal shedding and BV. The significant association of being PCR negative for *Prevotella spp*. and HIV RNA vaginal shedding is considered to be significant at random.

In an American study of 203 WLWH with BV diagnosed with Nugent score, herpes simplex virus, but not HPV, was associated with HIV RNA in cervicovaginal lavage samples [5]. However, all of these women had plasma HIV RNA ≥4000 copies/mL, and were thereby not applicable to our setting where WLWH had fully suppressed plasma HIV RNA levels [5].

It has previously been described that BV (diagnosed with Amsel’s criteria) induces HIV RNA vaginal shedding [28]. In 2013, Mitchell et al. [29] found an association between genital HIV RNA shedding and BV associated species (diagnosed using Nugent’s criteria) among 104 WLWH on ART.

The association between BV and HIV acquisition/transmission seems to be strong [30]. A meta-analysis assessing 25 different study populations from the US, Thailand and sub-Saharan Africa including almost 31,000 women, concluded that BV increases the risk of HIV acquisition by approximately 60% [3]. In this meta-analysis BV was diagnosed with Nugent’s criteria in 12/25 study populations and by clinical criteria only in 13/25 study populations [3]. The overall BV prevalence was 33%, ranging from 11% in women aged 20–35 years from the US to 70% in South African women with symptoms of STI’s. [3] In a sub-Saharan African study among 2236 sero-discordant couples, BV (diagnosed with Nugent’s criteria) was associated with a more than three-fold higher risk of HIV female-male transmission, after controlling for socio-demographic factors, sexual behaviour, male circumcision, STIs, pregnancy and plasma HIV RNA levels [4]. While a high prevalence of BV was also found in the present study, most patients were on suppressive ART with low vaginal viral loads, unlike most patients in the abovementioned studies, and we found no relation between BV status and vaginal HIV RNA shedding. Our findings emphasize the importance of ART, and support the treatment as prevention studies, where patients on ART are unlikely to transmit HIV [31].

In the current study, around 40% of women with symptoms from the lower abdomen were BV positive but we found no associations between having symptoms and BV. In line with this, an American study of 2888 women without gonorrhoea or trichomonas found a high prevalence of symptoms in both women with and without BV, and with no significant difference between the groups [32]. All BV diagnoses were made by using Nugent’s criteria.

Bacterial vaginosis is often asymptomatic, under-diagnosed and –treated [32].

Strengths of the present study are that all microscopy samples were analysed by the same observer and the nationwide inclusion, thereby giving a wider image of the female HIV-infected population in Denmark. Our cohort has previously been shown to include very few other STI’s, [12] which is why no further testing was made in the present study. Furthermore, the molecular methods allowed an objective determination of BV in the group with intermediate flora and had the potential to sub-classify BV according to the dominating species.A limitation may be the relatively small number of included patients, representing 14% of Denmark’s WLWH. Also, the inclusion and exclusion criteria might have led to the elimination of high-risk patients, as there were significantly less intravenous drug users included in the study, as well as more women on ART with lower HIV RNA counts and higher CD4 cell counts. (Thorsteinsson K, personal communication) However, there was no significant difference in ethnicity between the two groups. (Thorsteinsson K, personal communication). Finally, analysis for *Gardnerella vaginalis*, BVAB2, Eggerthella-like bacterium, and *Leptotrichia amnionii* could have been considered for analysis, however, these bacteria overlap significantly in their presence with the current selection [15], and it was considered to be too costly to include these additional species. Rather, it should be considered to re-analyse the samples using 16S rRNA gene based deep-sequencing to more thoroughly characterise the vaginal microbiota.

## Conclusion

BV is highly prevalent among well-treated women living with HIV in Denmark and more than 10% are positive for vaginal HIV RNA shedding. However, in this population, BV, herpes viridae and HPV do not predict vaginal HIV RNA shedding and, importantly, do thereby not seem to increase the risk of HIV transmission to partners or new-borns.

## Abbreviations

ART: Antiretroviral therapy
BV: Bacterial vaginosis
H_2_O_2_: Hydrogen Peroxide
HPV: Human papillomavirus
IQR: Interquartile range
NNRTI: Non-nucleoside reverse transcriptase inhibitor
OR: Odds ratio
PCR: Polymerase chain reaction
PI: Protease inhibitor
PLWH: People Living with HIV
SHADE: Study on HIV, cervical Abnormalities and infections in women in Denmark
STI: Sexually Transmitted Infection
WLWH: Women Living With HIV
